# Supplementary material for: Fluoxetine-induced alteration of murine gut microbial community structure: evidence for a microbial endocrinology-based mechanism of action responsible for fluoxetine-induced side effects
Source: PeerJ. 2019 Jan 9;7:e6199. doi: 10.7717/peerj.6199 (PMC6330042; doi:10.7717/peerj.6199)
Supplement: Table S1 [file peerj-07-6199-s001.docx]

**Table S1: Species richness and diversity estimators**

| **Animal*** | **Species observed** | **Chao (Richness)** | **ACE (Richness)** | **Shannon (Diversity)** | **Npshannon (Diversity)** | **Simpson (Evenness)** |
| --- | --- | --- | --- | --- | --- | --- |
| 5C D0 | 442 | 720.80 | 991.31 | 4.08 | 4.17 | 0.036 |
| 5C D15 | 533 | 796.65 | 944.99 | 4.63 | 4.71 | 0.023 |
| 5C D29 | 536 | 796.04 | 914.40 | 4.81 | 4.89 | 0.016 |
| 6C D0 | 438 | 639.11 | 860.41 | 4.12 | 4.20 | 0.037 |
| 6C D15 | 508 | 745.54 | 902.72 | 4.68 | 4.75 | 0.022 |
| 6C D29 | 538 | 748.99 | 753.49 | 4.67 | 4.75 | 0.023 |
| 7C D0 | 462 | 679.50 | 796.59 | 4.49 | 4.56 | 0.024 |
| 7C D15 | 484 | 615.06 | 638.90 | 4.74 | 4.80 | 0.018 |
| 7C D29 | 477 | 652.04 | 819.75 | 4.55 | 4.63 | 0.023 |
| 8C D0 | 443 | 614.27 | 589.84 | 4.67 | 4.72 | 0.020 |
| 8C D15 | 475 | 689.76 | 797.63 | 4.67 | 4.73 | 0.022 |
| 8C D29 | 478 | 667.57 | 676.00 | 4.51 | 4.59 | 0.025 |
| 13C D0 | 498 | 719.67 | 732.18 | 4.43 | 4.51 | 0.028 |
| 13C D15 | 502 | 679.00 | 710.92 | 4.32 | 4.40 | 0.038 |
| 13C D29 | 498 | 667.27 | 685.41 | 4.65 | 4.72 | 0.025 |
| 14C D0 | 494 | 618.01 | 645.70 | 4.59 | 4.66 | 0.024 |
| 14C D15 | 521 | 806.00 | 953.27 | 4.55 | 4.64 | 0.024 |
| 14C D29 | 476 | 673.73 | 829.17 | 4.53 | 4.60 | 0.026 |
| 15C D0 | 516 | 808.90 | 954.21 | 4.75 | 4.82 | 0.017 |
| 15C D15 | 530 | 749.61 | 725.33 | 4.79 | 4.86 | 0.018 |
| 15C D29 | 515 | 807.52 | 919.76 | 4.79 | 4.86 | 0.017 |
| 16C D0 | 517 | 653.77 | 681.01 | 4.75 | 4.82 | 0.021 |
| 16C D15 | 499 | 777.61 | 915.31 | 4.46 | 4.54 | 0.033 |
| 16C D29 | 457 | 680.75 | 799.17 | 4.33 | 4.41 | 0.029 |
| 19C D0 | 435 | 602.61 | 623.87 | 4.35 | 4.42 | 0.027 |
| 19C D15 | 454 | 598.22 | 758.57 | 4.13 | 4.21 | 0.055 |
| 19C D29 | 496 | 662.11 | 648.79 | 4.64 | 4.70 | 0.026 |
| 20C D0 | 471 | 678.00 | 644.32 | 4.53 | 4.60 | 0.025 |
| 20C D15 | 471 | 717.82 | 823.47 | 4.38 | 4.46 | 0.034 |
| 20C D29 | 462 | 703.67 | 810.38 | 4.61 | 4.67 | 0.020 |
| 1E D0 | 476 | 673.44 | 689.61 | 4.50 | 4.58 | 0.023 |
| 1E D15 | 475 | 682.13 | 677.67 | 4.35 | 4.43 | 0.033 |
| 1E D29 | 435 | 580.09 | 579.52 | 4.31 | 4.38 | 0.030 |
| 2E D0 | 410 | 571.80 | 575.87 | 4.24 | 4.31 | 0.038 |
| 2E D15 | 502 | 764.71 | 868.01 | 4.40 | 4.48 | 0.034 |
| 2E D29 | 536 | 766.00 | 875.29 | 4.84 | 4.91 | 0.017 |
| 3E D0 | 446 | 627.25 | 731.10 | 4.45 | 4.51 | 0.030 |
| 3E D15 | 458 | 669.14 | 739.83 | 4.45 | 4.52 | 0.036 |
| 3E D29 | 406 | 640.02 | 716.23 | 3.95 | 4.02 | 0.055 |
| 4E D0 | 510 | 751.38 | 849.15 | 4.75 | 4.81 | 0.020 |
| 4E D15 | 547 | 788.54 | 761.09 | 4.83 | 4.90 | 0.019 |
| 4E D29 | 463 | 659.09 | 755.74 | 4.45 | 4.52 | 0.028 |
| 9E D0 | 507 | 682.35 | 688.10 | 4.71 | 4.78 | 0.019 |
| 9E D15 | 457 | 592.14 | 597.69 | 4.34 | 4.41 | 0.039 |
| 9E D29 | 473 | 677.10 | 771.82 | 4.90 | 4.96 | 0.015 |
| 10E D0 | 515 | 672.00 | 687.02 | 4.98 | 5.04 | 0.014 |
| 10E D15 | 548 | 769.04 | 766.01 | 5.00 | 5.06 | 0.014 |
| 10E D29 | 528 | 750.58 | 735.42 | 4.77 | 4.84 | 0.022 |
| 11E D0 | 399 | 591.24 | 782.70 | 3.99 | 4.07 | 0.040 |
| 11E D15 | 517 | 765.18 | 929.06 | 4.69 | 4.76 | 0.019 |
| 11E D29 | 475 | 684.55 | 805.31 | 4.45 | 4.52 | 0.029 |
| 12E D0 | 506 | 738.48 | 820.51 | 4.59 | 4.65 | 0.034 |
| 12E D15 | 454 | 672.31 | 785.07 | 4.58 | 4.64 | 0.028 |
| 12E D29 | 464 | 684.65 | 797.73 | 4.31 | 4.39 | 0.033 |
| 17E D0 | 485 | 724.63 | 855.58 | 4.64 | 4.71 | 0.021 |
| 17E D15 | 498 | 679.18 | 703.75 | 4.52 | 4.60 | 0.024 |
| 17E D29 | 468 | 703.49 | 785.72 | 4.50 | 4.57 | 0.026 |
| 18E D0 | 462 | 613.76 | 650.41 | 4.33 | 4.41 | 0.030 |
| 18E D15 | 503 | 723.00 | 714.21 | 4.79 | 4.85 | 0.018 |
| 18E D29 | 507 | 754.78 | 871.82 | 4.51 | 4.59 | 0.025 |

* E: Fluoxtine treated animals, C: control group, D: day
