# Supplementary material for: Fluoxetine-induced alteration of murine gut microbial community structure: evidence for a microbial endocrinology-based mechanism of action responsible for fluoxetine-induced side effects
Source: PeerJ. 2019 Jan 9;7:e6199. doi: 10.7717/peerj.6199 (PMC6330042; doi:10.7717/peerj.6199)
Supplement: Table S2 [file peerj-07-6199-s002.docx]

| **OTU** | **Size** | **Phylum** | **Family** | **Genus** | **Relative abundance (%)** |
| --- | --- | --- | --- | --- | --- |
| Otu00001 | 53,574 | *Bacteroidetes* | *Rikenellaceae* | *Rikenellaceae_RC9_gut_group* | 4.72 |
| Otu00002 | 45,357 | *Bacteroidetes* | *Bacteroidales_S24-7_group* | *Bacteroidales_S24-7_group_ge* | 4.00 |
| Otu00003 | 40,269 | *Bacteroidetes* | *Bacteroidales_S24-7_group* | *Bacteroidales_S24-7_group_ge* | 3.55 |
| Otu00004 | 32,091 | *Bacteroidetes* | *Bacteroidales_S24-7_group* | *Bacteroidales_S24-7_group_ge* | 2.83 |
| Otu00005 | 26,731 | *Firmicutes* | *Lactobacillaceae* | *Lactobacillus apodemi* | 2.36 |
| Otu00006 | 26,513 | *Bacteroidetes* | *Bacteroidales_S24-7_group* | *Bacteroidales_S24-7_group_ge* | 2.34 |
| Otu00007 | 25,041 | *Bacteroidetes* | *Bacteroidaceae* | *Bacteroides acidifaciens* | 2.21 |
| Otu00008 | 24,586 | *Bacteroidetes* | *Bacteroidales_S24-7_group* | *Bacteroidales_S24-7_group_ge* | 2.17 |
| Otu00009 | 23,683 | *Firmicutes* | *Lachnospiraceae* | *Roseburia faecis* | 2.09 |
| Otu00010 | 22,977 | *Firmicutes* | *Lachnospiraceae* | *Lachnospiraceae_UCG-001* | 2.02 |
| Otu00011 | 22,675 | *Firmicutes* | *Lachnospiraceae* | *Lachnospiraceae_NK4A136_group* | 2.00 |
| Otu00012 | 21,941 | *Bacteroidetes* | *Bacteroidaceae* | *Bacteroides acidifaciens* | 1.93 |
| Otu00013 | 20,694 | *Bacteroidetes* | *Bacteroidales_S24-7_group* | *Bacteroidales_S24-7_group_ge* | 1.82 |
| Otu00014 | 19,396 | *Firmicutes* | *Lachnospiraceae* | *Lachnospiraceae_UCG-001* | 1.71 |
| Otu00015 | 19,332 | *Bacteroidetes* | *Bacteroidales_S24-7_group* | *Bacteroidales_S24-7_group_ge* | 1.70 |
| Otu00016 | 19,117 | *Firmicutes* | *Lachnospiraceae* | *Lachnospiraceae_NK4A136_group* | 1.68 |
| Otu00017 | 14,770 | *Firmicutes* | *Lactobacillaceae* | *Lactobacillus johnsonii* | 1.30 |
| Otu00018 | 13,434 | *Deferribacteres* | *Deferribacteraceae* | *Mucispirillum schaedleri* | 1.18 |
| Otu00019 | 13,141 | *Bacteroidetes* | *Bacteroidales_S24-7_group* | *Bacteroidales_S24-7_group_ge* | 1.16 |
| Otu00020 | 11,193 | *Firmicutes* | *Erysipelotrichaceae* | *Ileibacterium valens* | 0.99 |
| Otu00021 | 11,190 | *Firmicutes* | *Lachnospiraceae* | *Lachnospiraceae_NK4A136_group* | 0.99 |
| Otu00022 | 9,974 | *Bacteroidetes* | *Bacteroidales_S24-7_group* | *Bacteroidales_S24-7_group_ge* | 0.88 |
| Otu00023 | 8,944 | *Bacteroidetes* | *Rikenellaceae* | *Alistipes finegoldii* | 0.79 |
| Otu00024 | 8,816 | *Bacteroidetes* | *Rikenellaceae* | *Alistipes finegoldii* | 0.78 |
| Otu00025 | 8,673 | *Bacteroidetes* | *Rikenellaceae* | *Alistipes finegoldii* | 0.76 |
| Otu00026 | 8,179 | *Bacteroidetes* | *Rikenellaceae* | *Rikenellaceae_unclassified* | 0.72 |
| Otu00027 | 7,910 | *Firmicutes* | *Lactobacillaceae* | *Lactobacillus reuteri* | 0.70 |
| Otu00028 | 7,696 | *Firmicutes* | *Lachnospiraceae* | *Lachnospiraceae_unclassified* | 0.68 |
| Otu00029 | 7,367 | *Firmicutes* | *Lachnospiraceae* | *Lachnospiraceae_unclassified* | 0.65 |
| Otu00030 | 7,253 | *Firmicutes* | *Lachnospiraceae* | *Lachnospiraceae_unclassified* | 0.64 |
| Otu00031 | 6,854 | *Bacteroidetes* | *Bacteroidales_S24-7_group* | *Bacteroidales_S24-7_group_ge* | 0.60 |
| Otu00032 | 6,610 | *Firmicutes* | *Lachnospiraceae* | *Lachnospiraceae_unclassified* | 0.58 |
| Otu00033 | 6,361 | *Firmicutes* | *Clostridiaceae_1* | *Candidatus_Arthromitus* | 0.56 |
| Otu00034 | 6,273 | *Firmicutes* | *Lachnospiraceae* | *Lachnospiraceae_UCG-001* | 0.55 |
| Otu00035 | 6,173 | *Bacteroidetes* | *Bacteroidales_S24-7_group* | *Bacteroidales_S24-7_group_ge* | 0.54 |
| Otu00036 | 5,860 | *Firmicutes* | *Lachnospiraceae* | *Lachnospiraceae_unclassified* | 0.52 |
| Otu00037 | 5,748 | *Firmicutes* | *Lachnospiraceae* | *Lachnospiraceae_NK4A136_group* | 0.51 |
| Otu00038 | 5,715 | *Firmicutes* | *Lachnospiraceae* | *Lachnospiraceae_ge* | 0.50 |
| Otu00039 | 5,608 | *Bacteroidetes* | *Bacteroidales_S24-7_group* | *Bacteroidales_S24-7_group_ge* | 0.49 |
| Otu00040 | 5,578 | *Firmicutes* | *Ruminococcaceae* | *Ruminococcaceae_UCG-014* | 0.49 |
| Otu00041 | 5,533 | *Bacteroidetes* | *Bacteroidales_S24-7_group* | *Bacteroidales_S24-7_group_ge* | 0.49 |
| Otu00042 | 5,056 | *Firmicutes* | *Lachnospiraceae* | *Lachnospiraceae_unclassified* | 0.45 |
| Otu00043 | 4,958 | *Firmicutes* | *Lachnospiraceae* | *Lachnospiraceae_unclassified* | 0.44 |
| Otu00044 | 4,861 | *Firmicutes* | *Erysipelotrichaceae* | *Allobaculum* | 0.43 |
| Otu00045 | 4,739 | *Firmicutes* | *Lachnospiraceae* | *Roseburia faecis* | 0.42 |
| Otu00046 | 4,735 | *Firmicutes* | *Lachnospiraceae* | *Lachnoclostridium scindens* | 0.42 |
| Otu00047 | 4,675 | *Bacteroidetes* | *Rikenellaceae* | *Alistipes finegoldii* | 0.41 |
| Otu00048 | 4,479 | *Firmicutes* | *Lachnospiraceae* | *Lachnospiraceae_NK4A136_group* | 0.39 |
| Otu00049 | 4,478 | *Firmicutes* | *Lachnospiraceae* | *Lachnospiraceae_NK4A136_group* | 0.39 |
| Otu00050 | 4,408 | *Firmicutes* | *Ruminococcaceae* | *Ruminiclostridium_9* | 0.39 |
