# Supplementary material for: Fluoxetine-induced alteration of murine gut microbial community structure: evidence for a microbial endocrinology-based mechanism of action responsible for fluoxetine-induced side effects
Source: PeerJ. 2019 Jan 9;7:e6199. doi: 10.7717/peerj.6199 (PMC6330042; doi:10.7717/peerj.6199)
Supplement: Table S3 [file peerj-07-6199-s003.docx]

| **Query id (OTU)** | **subject id (miBC 16S rRNA genes), GenBank accession no.** | **% identity** | **alignment length** | **e-value** | **bit score** |
| --- | --- | --- | --- | --- | --- |
| Otu00005 | AJ871178.1_*Lactobacillus_apodemi*,_type_strain_ASB1T | 100 | 253 | 3.49E-134 | 468 |
| Otu00007 | KR364741.1_*Bacteroides_caecimuris*_strain_I48 | 95.6 | 253 | 7.72E-116 | 407 |
| Otu00008 | KR364784.1_*Muribaculum_intestinale*_strain_YL27 | 95.7 | 254 | 2.77E-115 | 405 |
| Otu00012 | AB021164.1_*Bacteroides_acidofaciens*_strain_A40 | 100 | 253 | 3.49E-134 | 468 |
| Otu00017 | KU196088.1_*Lactobacillus_johnsonii*_strain_DSM_100219 | 100 | 253 | 3.49E-134 | 468 |
| Otu00023 | KF809885.1_*Alistipes_sp._CC-5826*-wt-bac | 95.3 | 253 | 3.59E-114 | 401 |
| Otu00024 | KF809885.1_*Alistipes_sp._CC-5826*-wt-bac | 95.7 | 253 | 7.72E-116 | 407 |
| Otu00027 | KR364775.1_*Lactobacillus_reuteri*_strain_M-6220-5A | 100 | 253 | 3.49E-134 | 468 |
| Otu00028 | KC311366.2_*Murimonas_intestini*_strain_SRB-530-5-H | 95.3 | 253 | 3.59E-114 | 401 |
| Otu00029 | KR364760.1_*Cuneatibacter_caecimuris*_strain_BARN-424-CC-10 | 95.3 | 256 | 3.59E-114 | 401 |
| Otu00030 | KR364770.1_*Muricomes_intestini*_strain_2PG-424-CC-1 | 95.7 | 253 | 7.72E-116 | 407 |
| Otu00032 | KR364760.1_*Cuneatibacter_caecimuris*_strain_BARN-424-CC-10 | 95.3 | 254 | 3.59E-114 | 401 |
| Otu00039 | KR364784.1_*Muribaculum_intestinale*_strain_YL27 | 96.4 | 252 | 1.28E-118 | 416 |
| Otu00041 | KR364784.1_*Muribaculum_intestinale*_strain_YL27 | 100 | 252 | 1.25E-133 | 466 |
| Otu00046 | KR364770.1_*Muricomes_intestini*_strain_2PG-424-CC-1_ | 95.3 | 253 | 3.59E-114 | 401 |
| Otu00050 | KF447772.1_*Flintibacter_butyricus*_strain_BLS21 | 98.0 | 253 | 7.61E-126 | 440 |
| Otu00061 | KF447772.1_*Flintibacter_butyricus*_strain_BLS21 | 99.6 | 253 | 1.63E-132 | 462 |
| Otu00070 | KC311366.2_*Murimonas_intestini*_strain_SRB-530-5-H | 95.3 | 253 | 3.59E-114 | 401 |
| Otu00080 | GQ456204.2_*Bacteroides_sartorii*_JCM_17136_=_DSM_21941_strain_A-C2-0_16S | 98.4 | 253 | 1.64E-127 | 446 |
| Otu00081 | KR364746.1_*Blautia_caecimuris*_strain_SJ18 | 95.7 | 254 | 7.72E-116 | 407 |
| Otu00087 | KR364784.1_*Muribaculum_intestinale*_strain_YL27 | 95.2 | 252 | 1.29E-113 | 399 |
| Otu00093 | KR364773.1_*Flavonifractor_plautii*_strain_YL31 | 96.8 | 253 | 7.67E-121 | 424 |
| Otu00100 | KR364760.1_*Cuneatibacter_caecimuris*_strain_BARN-424-CC-10 | 95.7 | 255 | 7.72E-116 | 407 |
