# Supplementary material for: Fluoxetine-induced alteration of murine gut microbial community structure: evidence for a microbial endocrinology-based mechanism of action responsible for fluoxetine-induced side effects
Source: PeerJ. 2019 Jan 9;7:e6199. doi: 10.7717/peerj.6199 (PMC6330042; doi:10.7717/peerj.6199)
Supplement: Table S4 — Significantly different OTUs are shown for the 100 most abundant OTUs. [file peerj-07-6199-s004.docx]

| **OTU** | **Median relative abundance control (%)** | **Median relative abundance Fluoxetine (%)** | **LDA score (log 10)** | **p-value** | **Taxonomy** |
| --- | --- | --- | --- | --- | --- |
| Otu00006 | **1.89** | 1.22 | 3.71 | 0.01 | *Bacteroidales_S24-7_*group |
| Otu00017 | **1.13** | 0.15 | 3.53 | 0.01 | *Lactobacillus johnsonii* |
| Otu00019 | **1.15** | 0.79 | 3.28 | 0.02 | *Bacteroidales_S24-7_*group |
| Otu00024 | 0.31 | **0.90** | 3.44 | 0.02 | *Alistipes finegoldii* |
| Otu00028 | **0.52** | 0.11 | 3.51 | 0.02 | *Lachnospiraceae_unclassified* |
| Otu00032 | 0.13 | **0.59** | 3.37 | 0.01 | *Lachnospiraceae_unclassified* |
| Otu00038 | 0.14 | **0.55** | 3.27 | 0.003 | *Lachnospiraceae* |
| Otu00039 | **0.58** | 0.19 | 3.23 | 0.0007 | *Bacteroidales_S24-7_*group |
| Otu00040 | **0.49** | 0.05 | 3.40 | 0.001 | *Ruminococcaceae_UCG-014* |
| Otu00041 | **0.51** | 0.26 | 3.11 | 0.01 | *Bacteroidales_S24-7_*group |
| Otu00042 | **0.03** | 0.00 | 3.33 | 0.007 | *Lachnospiraceae_unclassified* |
| Otu00045 | 0.04 | **0.26** | 3.21 | 0.005 | *Roseburia faecis* |
| Otu00046 | 0.33 | **0.56** | 3.06 | 0.0005 | *Lachnoclostridium scindens* |
| Otu00048 | **0.21** | 0.02 | 3.30 | 0.0004 | *Lachnospiraceae_NK4A136_*group |
| Otu00062 | 0.17 | **0.36** | 3.08 | 0.01 | *Anaerotruncus* |
| Otu00066 | **0.24** | 0.12 | 3.22 | 0.002 | *Bacteroidales_S24-7_*group |
| Otu00069 | **0.17** | 0.05 | 3.24 | 0.002 | *Roseburia faecis* |
| Otu00074 | 0.12 | **0.31** | 2.96 | 0.01 | *Roseburia faecis* |
| Otu00086 | 0.07 | **0.19** | 2.96 | 0.002 | *Lachnospiraceae_unclassified* |
| Otu00092 | **0.14** | 0.02 | 2.83 | 0.01 | *Lachnospiraceae_unclassified* |
| Otu00093 | 0.12 | **0.20** | 2.64 | 0.03 | *Ruminococcaceae_unclassified* |
