# Supplementary material for: Fluoxetine-induced alteration of murine gut microbial community structure: evidence for a microbial endocrinology-based mechanism of action responsible for fluoxetine-induced side effects
Source: PeerJ. 2019 Jan 9;7:e6199. doi: 10.7717/peerj.6199 (PMC6330042; doi:10.7717/peerj.6199)
Supplement: Table S5 — Significantly different genera are shown for the 50 most abundant genera. [file peerj-07-6199-s005.docx]

| **Genus** | **Median relative abundance control (%)** | **Median relative abundance fluoxetine (%)** | **LDA score (log 10)** | **p-value** |
| --- | --- | --- | --- | --- |
| *Bacteroidales_S24-7_group* | **28.91** | 21.44 | 4.61 | 0.03 |
| *Lachnospiraceae_UCG-001* | 3.32 | **5.19** | 4.17 | 0.04 |
| *Ruminococcaceae_UCG-014* | **1.58** | 0.95 | 3.52 | 0.004 |
| *Mollicutes_RF9* | **1.01** | 0.60 | 3.25 | 0.01 |
| *Lachnospiraceae uncultured* | 0.73 | **1.13** | 3.23 | 0.01 |
| *Lachnoclostridium* | 0.49 | **0.92** | 3.23 | 0.004 |
| *Anaerotruncus* | 0.40 | **0.60** | 3.23 | 0.02 |
| *Ruminiclostridium_5* | 0.34 | **0.46** | 2.91 | 0.03 |
| *Coriobacteriaceae_unclassified* | 0.11 | **0.17** | 2.40 | 0.02 |
| *Lachnospiraceae_UCG-006* | 0.05 | **0.13** | 2.68 | 0.0001 |
